# The lncrnas: innovative multifunctional players of drug resistance in colorectal cancer

**DOI:** 10.1186/s12935-025-03951-3

**Published:** 2025-11-23

**Authors:** Hossein Maghsoudi, Motahareh Rasoulzadeh, Maryam Abbastabar, Ahmad Fazilat, Farhad Sheikhnia, Bita Azizzadeh, Mohamad Valilo, Maryam Majidinia

**Affiliations:** 1 Student Research Committee, Urmia University of Medical Sciences, Urmia, Iran; 2 Department of Clinical Biochemistry, School of Medicine, Urmia University of Medical Sciences, Urmia, Iran; 3 Department of Toxicology and Pharmacology, School of Pharmacy, Shahid Beheshti University of Medical Sciences, Tehran, Iran; 4 Department of Clinical Biochemistry, School of Medicine, Babol University of Medical Sciences, Babol, Iran; 5 Motamed Cancer Institute, Breast Cancer Research Center, ACECR, Tehran, Iran; 6 Solid Tumor Research Center, Cellular and Molecular Medicine Research Institute, Urmia University of Medical Sciences, Urmia, Iran

**Keywords:** Colorectal cancer, Drug resistance, LncRNAs

## Abstract

Colorectal cancer (CRC) ranks as the third most prevalent malignancy and the second leading cause of cancer-related mortality globally. Despite substantial advancements in CRC therapies over recent decades, drug resistance continues to pose a significant challenge in effective treatment. This comprehensive review investigates the role of long non-coding RNAs (lncRNAs) in the emergence of drug resistance in CRC. Specifically, we analyze the functions of lncRNAs, including HOTAIR and MALAT1, in key processes such as apoptosis, DNA damage response and repair, cancer stem cell regulation, and drug metabolism. By examining the structural and biogenetic characteristics of lncRNAs, this review elucidates their specific contributions to drug resistance. We provide novel insights into the therapeutic potential of targeting lncRNAs to mitigate drug resistance in CRC, thereby offering new perspectives on treatment strategies. Ultimately, this review highlights the clinical implications of lncRNA research, emphasizing their potential as targets for innovative therapeutic interventions in colorectal cancer.

**Graphical abstract:**

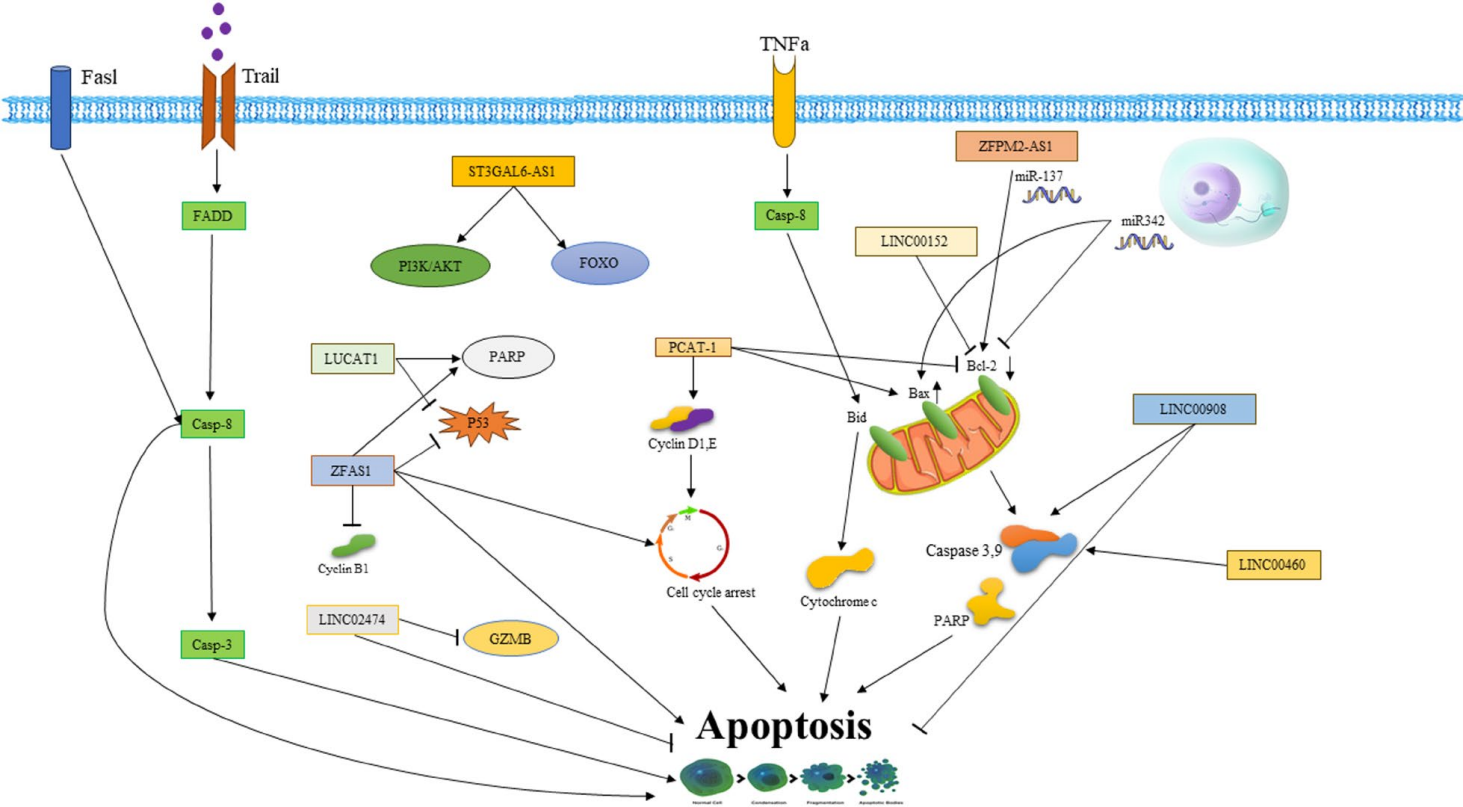

**Supplementary Information:**

The online version contains supplementary material available at 10.1186/s12935-025-03951-3.

## Introduction

Colorectal cancer (CRC) ranks as the third most frequently diagnosed malignancy and stands as the second leading cause of cancer-related mortality worldwide [1]. Projections indicate that by 2030, there will be an estimated 2.5 million new cases and 1.1 million deaths attributable to CRC [2]. The five-year survival rate is significantly influenced by the stage at which the disease is diagnosed. Patients with stage I CRC exhibit a survival rate exceeding 90%, whereas those with stage IV CRC have a survival rate of approximately 10%. Consequently, early detection of the disease is of paramount importance. In the absence of timely screening, treating advanced-stage CRC becomes exceedingly challenging [3].

The conventional treatment modalities for CRC include surgical intervention, chemotherapy, and radiotherapy [4]. Despite substantial advancements in therapeutic interventions, the emergence of drug resistance remains a formidable obstacle to effective treatment strategies [5, 6]. Approximately 80% of patients responding to epidermal growth factor receptor inhibitors (anti-EGFR) therapies in metastatic CRC eventually develop drug resistance [7]. Furthermore, acquired resistance to therapy occurs in 90% of patients with metastatic cancer [8]. This resistance markedly diminishes the efficacy of treatments such as chemotherapy, molecularly targeted therapy, and immunotherapy [9].

Oxidative stress, arising from an imbalance between oxidants and antioxidants, leads to modifications in DNA and proteins, in addition to lipid peroxidation. This phenomenon is intimately linked with the pathogenesis of CRC and the development of drug resistance [10–12] Drug resistance in CRC can be broadly categorized into intrinsic and acquired resistance. Intrinsic resistance is commonly observed in the initial stages of treatment or during the preliminary phases of clinical trials. Acquired resistance, however, can develop through distinct molecular mechanisms and is unique to each therapeutic intervention [13]. CRC cells may exhibit intrinsic resistance due to genetic mutations, altered signaling pathways, or overexpression of efflux transporters that reduce intracellular drug accumulation [14]. A deeper understanding of both intrinsic and acquired resistance mechanisms would significantly enhance drug development efforts.

Recently, the significance of the tumor microenvironment (TME) in CRC has garnered considerable attention, prompting a comprehensive examination of clinical trials to evaluate immune-cell infiltration as prognostic and predictive indicators [15, 16]. The TME, which comprises various cell types such as immune cells, fibroblasts, and endothelial cells, plays a crucial role in tumor progression and response to therapy [17]. A growing body of evidence elucidates that epigenetic regulation constitutes a pivotal mechanism underpinning both intrinsic and acquired drug resistance in CRC. Non-coding RNAs (ncRNAs), including long non-coding RNAs (lncRNAs) and microRNAs (miRNAs), are now recognized for their roles in various biological functions, both physiological and pathological, including drug resistance [6, 18–22]. These ncRNAs can modulate gene expression and signaling pathways, thereby influencing the sensitivity of cancer cells to therapeutic agents.

Understanding the role of lncRNAs in drug resistance mechanisms could unveil novel therapeutic targets and strategies to overcome resistance and improve treatment outcomes for CRC patients. Future research should focus on elucidating the complex interactions between lncRNAs and other molecular players within the TME. Additionally, the development of lncRNA-based therapeutics holds promise for enhancing the efficacy of existing treatments and potentially reversing drug resistance in CRC. By integrating insights from molecular biology, genomics, and clinical research, we can pave the way for more effective and personalized therapeutic approaches for CRC patients.

A comprehensive literature search was conducted to identify relevant studies published between 2010 and 2024. The databases searched included PubMed, Google Scholar, Scopus, and Web of Science. The following keywords were used in various combinations: “colorectal cancer”, “CRC”, “drug resistance”, “long non-coding RNAs”, and “lncRNAs.”

## CRC treatments

CRC necessitates a comprehensive therapeutic approach, with diverse treatment modalities often combined to enhance efficacy [23, 24]. Surgery remains the primary treatment for localized cancer, involving the removal of the tumor and lymph nodes [25]. Minimally invasive techniques, such as laparoscopic and robotic-assisted surgeries, reduce complications and promote faster recovery [26]. For advanced disease, multivisceral resections and cytoreductive surgery with hyperthermic intraperitoneal chemotherapy (HIPEC) may be considered [27]. Adjuvant chemotherapy, administered post-surgery, reduces the risk of recurrence. Common protocols include 5-fluorouracil (5-FU)-based combinations like FOLFOX [folinic acid (leucovorin), 5-FU, and oxaliplatin] and CAPOX (XELOX) [capecitabine (an oral prodrug of 5-FU) with oxaliplatin/OXA], which improve survival rates, particularly in stage III colon cancer. For metastatic CRC, systemic chemotherapy with FOLFOX or FOLFIRI [folinic acid, fluorouracil, and irinotecan] combined with targeted agents is standard. Oxaliplatin and irinotecan, used with fluoropyrimidines, are employed in various combinations to balance effectiveness and manage toxicity [28].

Targeted therapies have emerged from identifying specific molecular alterations in CRC. Monoclonal antibodies targeting EGFR, such as cetuximab and panitumumab, are effective in wild-type KRAS tumors. Anti-angiogenic agents like bevacizumab inhibit vascular endothelial growth factor (VEGF) activity, which is crucial in metastatic cases [29, 30]. Immunotherapy, particularly immune checkpoint inhibitors like pembrolizumab and nivolumab, has revolutionized cancer treatment. These therapies are effective in patients with microsatellite instability-high (MSI-H) or mismatch repair-deficient (dMMR) tumors, underscoring the importance of biomarker-guided strategies [31, 32].

### Complications associated with standard care for CRC

Standard care for CRC typically involves a combination of surgery, chemotherapy, and radiation therapy, but these treatments can lead to several complications. Surgical complications, particularly from colorectal anastomosis, can include bleeding, dehiscence (wound separation), leakage, strictures (narrowing of the bowel), and fistulas (abnormal connections between organs), along with risks of infection, bowel obstruction, and damage to surrounding organs [33]. Chemotherapy and radiation therapy can cause side effects such as nausea, vomiting, diarrhea, fatigue, neutropenia and increased susceptibility to infections, with long-term complications including neuropathy (nerve damage), cardiotoxicity (heart damage), and secondary cancers [34, 35]. Despite advancements in treatment, persistent failures in CRC care are often due to distant metastasis, which significantly reduces overall survival rates, and cancer drug resistance, where intrinsic resistance renders cancer cells insensitive to drugs even before treatment begins, leading to high recurrence rates and treatment failures [36, 37].

## Novel therapeutic strategies for combating drug resistance in CRC

To improve outcomes and reduce mortality rates, innovative research methods such as the combined administration of chemical drugs and commercialized medications with established effects, including drug repurposing (e.g., nonsteroidal anti-inflammatory drugs [NSAIDs], metformin, dichloroacetate, enalapril, ivermectin, bazedoxifene, melatonin, and S-adenosylmethionine), as well as natural herbal compounds (e.g., polyphenols, terpenoids, quinones, alkaloids, and sterols), have been explored [38–43]. Additionally, gene therapy, an emerging technology, has been extensively investigated as a means to reverse chemoresistance. This includes the utilization of ribozymes, RNA interference, CRISPR/Cas9, epigenetic therapy, antisense oligonucleotides, and noncoding RNAs, introducing a novel genetic dimension to this field. Furthermore, protein inhibitors are employed as countermeasures at the protein level, exerting their effects through direct interaction with specific proteins, such as EGFR inhibitors, sphingosine-1-phosphate receptor 2 (S1PR2) inhibitors, and DNA methyltransferase inhibitors [44–48]. It is widely recognized that conventional drug delivery systems possess various limitations, including low bioavailability and cytotoxicity, which contribute to the development of drug resistance (Table 1). In contrast, novel drug delivery systems, such as nanocarriers, liposomes, exosomes, and hydrogels, have the potential to overcome these drawbacks associated with traditional delivery methods. These innovative systems enable the direct transportation of therapeutic molecules to the precise tumor site, thus playing a crucial role in reversing chemoresistance [43, 49, 50].

**Table 1:** Drug repurposing and Natural herbal medicine applied in the reversal of chemoresistance in CRC

Drug	Chemical drugs	type of study	mechanisms	Reference
Aspirin: Nonsteroidal anti-inflammatory drugs	5-FU	in vitro: SW480, SW620 Invivo	decrease of 5-FU-induced NF-κB activation	[39]
	VCR	in vitro: CAF-like cells	decrease of TGF-βs and IL-6	[40]
Ibuprofen: Nonsteroidal anti-inflammatory drugs	VCR	in vitro: CAF-like cells	decrease of TGF-βs and IL-6	[40]
NS-398: Nonsteroidal anti-inflammatory drugs	VCR	in vitro: HCT-8	decrease of MDR1, P-gp and p‐c‐Jun	[52]
Metformin: The first-line treatment for type 2 diabetes mellitus	CDDP	in vitro: SW480, SW620	decrease of ROS production, inhibition of PI3K/Akt signaling pathway	[42]
	CPT-11	in vitro: HCT116 SW480	Block cell cycle in G1 and S phases	[41]
	5-FU	in vitro: SNU-C5	decrease of DNA replication and NF-κB, increased AMPK	[53]
	5-FU	in vitro: HCT116	decrease of stemness, inhibited EMT and Wnt3a/β-catenin pathway	[54]
Dichloroacetate: Metabolic diseases	5-FU	in vitro: HCT-8 in vivo	decrease of p53/miR-149-3p/PDK2 glucose metabolic pathway	[55]
	L-OHP	in vitro: HCT-116 in vivo	Activation of the AMPK-mTOR pathway	[56]
Enalapril: Antihypertensive and anti-heart failure	5-FU	in vitro: HCT116 SW620 in vivo	decrease of NF-κB/STAT3-regulated proteins (c-Myc, Cyclin D1, MMP-9, MMP-2, VEGF, Bcl-2, and XIAP)	[57]
Ivermectin: Antiparasitic drug	VCR	in vitro: HCT-8 in vivo	decrease of P-gp, EGFR, ERK/Akt/NF-κB signal	[58]
Bazedoxifene: Third-generation selective estrogen receptor modulator and novel IL‐6/GP130 target inhibitor	5-FU	in vitro: HT29, SW480, LOVO, HCT116 in vivo	Inhibition of IL-6/GP130 signaling pathway and phosphorylation of AKT, ERK and STAT3	[59]
Melatonin	5-FU	in vitro: HCT116 SW480	Increase of miR-215-5p and decrease of TYMS	[60]
S-Adenosylmethionine: Nutritional supplement	5-FU	in vitro: HCT116, LOVO	decrease of P-gp and activation of NF-κB	[61]
Vitamin C: Dietary supplements	L-OHP	in vitro: C2BBe1, WiDr	decrease of BAX/BCL2 ratio	[62]
Vitamin D: Dietary supplements	5-FU	in vitro: CBS, Moser, Caco-2	decrease of TS and surviving in aCaSR-dependent manner	[63]
1α,25-dihydroxyvitamin D3 Dietary supplements	5-FU/CPT‐11	in vitro: MIP101	Increase of vitamin D receptor (VDR)	[64]
Dihydromyricetin: Polyphenols	L-OHP, VCR	in vitro: HCT116, HCT8	Inhibit MRP2 expression and its promoter activity by preventing NF-κB‐Nrf2 signaling	[65]
Curcumin: Polyphenols	5-FU	in vitro: HCT116, HCT8	Inhibition of proliferation, inducement of apoptosis, block of G0/G1 phase and expression of TET1 and NKD2; inhibit proliferation, increase apoptosis, downregulate P-gp and HSP‐27	ADDIN EN.CITE [66, 67]
Resveratrol: Polyphenols	5-FU, L‐OHP	in vitro: HCT116, SW480	Inhibiting EMT via up-regulation of intercellular junctions and down‐regulation of NF‐κB pathway; downregulating MDR1, inhibiting NF‐kB pathway and the transcriptional activity of CRE	[68, 69]
β-elemene: Terpenoids	5-FU	in vitro: HCT116	Inhibit proliferation, induce pro-death autophagy and Cyclin D3‐dependent cycle arrest	[70]
Atractylenolide II: Terpenoids	5-FU, mitomycin, adriamycin, CDDP	in vitro: Lovo, SW480	Inhibit cell proliferation and alleviate chemoresistance	[71]
Emodin: Quinones	5-FU	in vitro: SW480	Inhibit proliferation, invasion, migration, and induce cell apoptosis and downregulate PI3K/Akt pathway	[72]
Tanshinone IIA: Quinones	L-OHP	in vitro: SW480	Decrease the levels of Bcl-2, p‐Akt and p‐ERK, and increase the levels of Bax and active caspase 3 by inhibiting ERK/Akt Signaling Pathway	[73]
Hypericin: Quinones	L-OHP	in vitro: HCT116, HCT8	Downregulation of MRP2 level, GSH-related detoxification and NER‐mediated DNA repair mediated by ROS	[74]
Evodiamine: Alkaloids	L-OHP	in vitro: HCT116	Inhibit cell growth, induce apoptosis, suppress the expression of ABCG2 and inhibit p50/p65 NF-κB Pathway	[75]
Ginsenoside Rh2: Sterols	5-FU	in vitro: HCT8, LOVO	Inhibit cell proliferation and migration, induce apoptosis, and decrease the expression of MRP1, MDR1, LRP and GST	[76]
β-Sitosterol: Sterols	L-OHP	in vitro: HCT116	Suppress BCRP, activate p53, and enhance apoptosis	[77]
Salvianolic acid B: phenolic acid	VCR	in vitro: HCT8	Increase ROS levels, promote apoptosis and downregulate the expression of P-gp	[78]

ABCB1, ATP binding cassette subfamily B member 1; ABCC5, ATP-binding cassette subfamily C member 5; ADAM17, A disintegrin and metalloproteinase 17; AKT, protein kinase B; ALDH1A3, aldehyde dehydrogenase 1 family, member A3; AOM, azoxymethane; AP‐1, activator protein‐1; ASOs, antisense oligonucleotides; anti‐miR‐19a, antisense oligonucleotide of miR‐19a; ATG10, autophagy‐related 10BCRP, breast cancer resistance protein; CAC1, CDK2‐associated cullin domain 1; CCAT1, colon cancer‐associated transcript 1; CCND2, cyclin D2; CDDP, cisplatin; CES‐2, carboxylesterase‐2; CircRNAs, circular RNAs; CSPP1, centrosome and spindle pole associated protein 1; CXCR4, C‐X‐C Motif chemokine receptor 4; DNMT, DNA methyltransferase; DOT1L, disruptor of telomeric silencing 1‐like; DOX, doxorubicin; EGFR, epidermal growth factor receptor; ERK, extracellular regulated protein kinases; DSS, dextran sodium sulfate; FKBPs, FK506‑binding proteins; FOLFOX, folinic acid, fluorouracil, and oxaliplatin; FOXM1, Forkhead box protein M1; 5‐FU, 5‐fluorouracil; FZD7, frizzled‐7; γ‐GCS, γ‐glutamylcysteine synthetase; GDPD5, glycerophosphodiester phosphodiesterase domain containing 5; GEM, gemcitabine; GOLPH3, golgi phosphoprotein 3; HCPT, hydroxycamptothecin; HDAC2, histone deacetylase 2; HIF‐1α, hypoxia inducible factor‐1α; KLK11, Kallikrein 11; KLF12, Krüppel‐like factor 12; L‐OHP, oxaliplatin; LncRNAs, long non‐coding RNAs; MDR, multidrug resistance; MDR1, multidrug resistance protein 1; METTL1, methyltransferase‐like 1; METTL3, methyltransferase‐like 3; m7G, 7‐methylguanosine; MiRNAs, micro RNAs; MRP, multidrug resistance‐associated protein; MSI1, musashi1; mTOR, mammalian target of rapamycin; NFIX, nuclear factor I/X; p‐AKT, phosphorylated AKT; P‐gp, P‐glycoprotein; PI3K, phosphatidylinositol 3 kinase; PPP, pentose phosphate pathway; PTEN, phosphatase and tensin homolog deleted on chromosome 10; PTX, paclitaxel; RBPJ, recombination signal binding protein for immunoglobulin Kappa J region; RBX2, RING box protein 2; RNAi, RNA interference; Rz, ribozyme; RRAGB, Ras‐related GTP binding protein B; SH3GL1, Src homology 3 (SH3)‐domain GRB2‐like protein 1; shRNA, short hairpin RNA; S6K1, S6 kinase 1; TALDO1, transaldolase 1; TIAM1, T‐lymphoma invasion and metastasis‐inducing protein‐1; TRIM67, tripartite motif‐containing 67; TYMS, thymidylate synthase; Ufd1, ubiquitin fusion‐degradation 1‐like protein; VP‐16, etoposide; Yap, yes‐associated protein; ABCG2, ATP‐binding cassette superfamily G member 2; BCRP, breast cancer resistance protein; CAFs, cancer‐associated fibroblasts; CDDP, cisplatin; CSCs, colorectal cancer stem cells; CRC, colorectal cancer; CRE, cAMP‐responsive element; EMT, epithelial‐mesenchymal transition; GSH, glutathione; GST, glutathione S‐transferase; Hsp27, heat shock protein 27; LRP, lung resistance‐related protein; MRP1, multidrug resistance‐associated protein 1; MRP 2, multidrug resistance‐associated protein 2; ncRNAs, noncoding RNAs; NER, nucleotide excision repair; NF‐κB, nuclear factor‐kappa B; NKD2, naked cuticle homolog 2; Nrf2, nuclear factor E2‐related factor 2; NSAIDs, nonsteroidal anti‐inflammation drugs; PCD, programmed cell death; p‐ERK, phosphorylated ERK; ROS, reactive oxygen species; siRNAs, small interfering RNAs; TAMs, tumor‐associated macrophages; TET1, ten‐eleven translocation; TGF‐β2, tumor growth factor‐β2; VCR, vincristine

## Molecular mechanisms of drug resistance

**Fig. 1:**
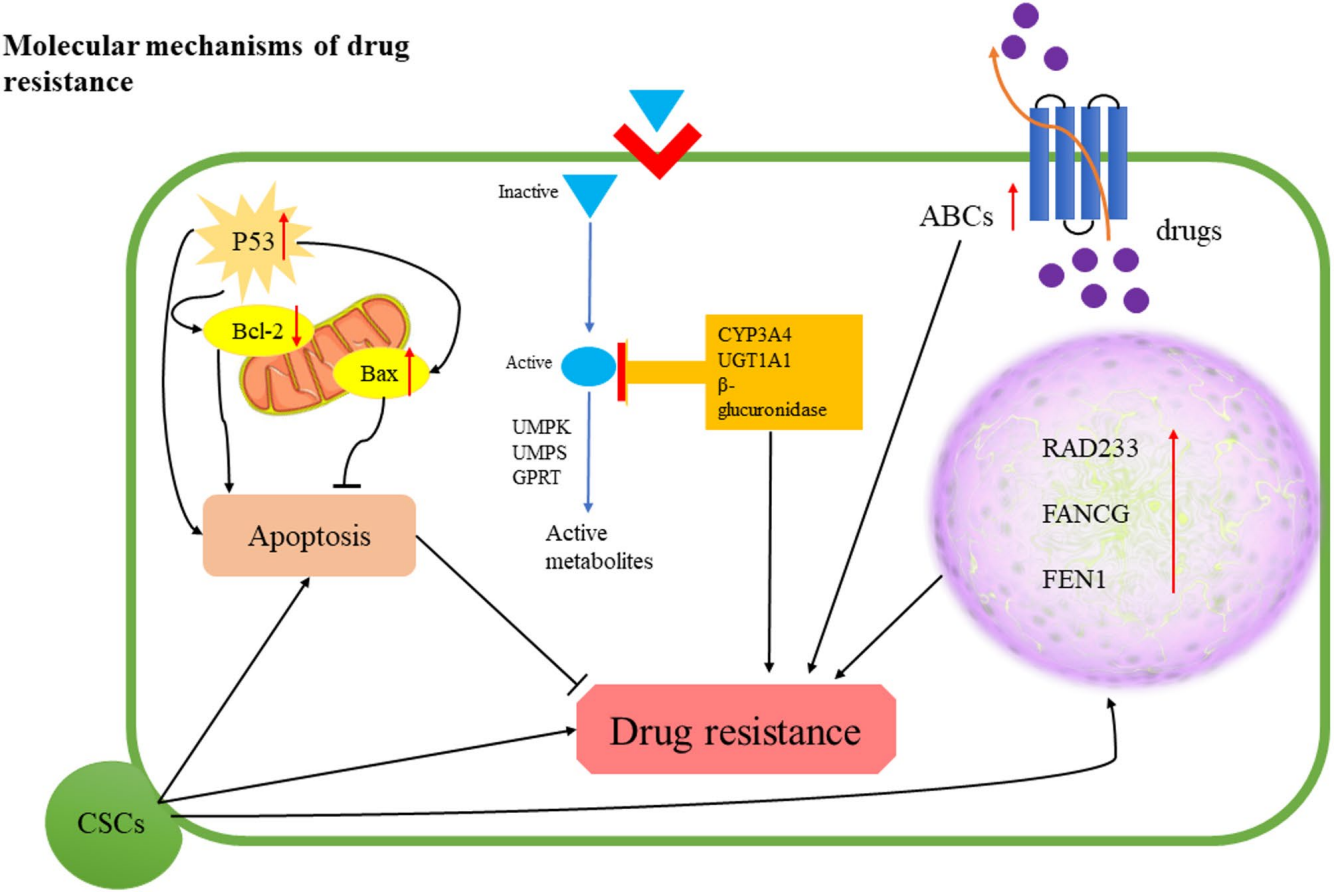
Molecular mechanisms of drug resistance in cancer cells

The development of drug-resistant cancer cells is influenced by a multitude of molecular mechanisms, highlighting the involvement of cellular deregulation and molecular factors (Fig. 1). Key predictors in this context include drug metabolism and transporters, cancer stem cells (CSCs), DNA damage response and repair, epithelial-mesenchymal transition (EMT), and apoptosis [77, 78].

### Apoptosis

When a drug effectively targets its intended molecules, the ideal outcome is the induction of programmed cell death, or apoptosis. Dysfunction in apoptotic pathways is closely associated with tumor drug resistance [79].

Resistance to programmed cell death is a prominent feature observed in malignant tumors [80, 81]. Apoptosis can be initiated through two distinct signaling pathways: the death receptor pathway and the mitochondrial pathway. Both pathways ultimately lead to the activation of caspase-3, a crucial protein in the apoptotic process [82]. Consequently, any aberrations in these signaling pathways can enhance a tumor’s resistance to therapy by impeding the execution of apoptosis.

Members of the Bcl-2 family regulate the intrinsic pathway of apoptosis, including anti-apoptotic proteins such as Bcl-2, Bcl-XL, and McL-1, as well as pro-apoptotic molecules such as Bax and BH3-only proteins [83]. Dysregulation of these complex processes can lead to evasion of apoptosis and drug resistance in tumors [84]. Preclinical investigations indicate that the resistance to 5-FU is associated with elevated levels of Bcl-2 and Bax expression in CRC. Furthermore, a reduction or absence of Bax expression has been linked to heightened resistance of CRC to both 5-FU and OXA. Hence, targeting the Bcl-2 protein family presents a potentially effective strategy for overcoming drug resistance [71, 85].

### Inhibitor of apoptosis (IAP) family

The IAP family plays a pivotal role in regulating the programmed cell death pathway. This family can impede the activation of caspases and exert a negative influence on the apoptosis pathway [86]. The IAP family primarily consists of neuronal IAP (NIAP), cellular IAP1 (cIAP1), cellular IAP2 (cIAP2), X chromosome-linked IAP (XIAP), and survivin [87]. Preclinical investigations have demonstrated that XIAP can enhance the resistance of CRC cells to apoptosis induced by various tumor treatments [88, 89].

Chemotherapeutic drugs induce DNA damage, triggering apoptosis in CRC cells. The tumor suppressor gene P53 plays a crucial role in this process. Mutations in P53 can interfere with DNA damage-induced cell cycle arrest and impede tumor cell apoptosis, contributing to chemotherapy resistance across various solid tumors, including CRC [90, 91]. Several clinical studies have substantiated that CRC patients with wild-type P53 benefit from 5-FU treatment, whereas those with mutant P53 face a less favorable prognosis during adjuvant chemotherapy [92–94].

### DNA damage response and repair

The DNA repair capacity (DRC) of tumor cells is implicated in resistance to various cancer treatments, such as chemoradiotherapy, targeted therapy, and immunotherapy [95]. Chemotherapeutic agents like cisplatin/CDDP and 5-FU, exert their anti-cancer effects by inducing DNA lesions in malignant cells. This activation of DNA damage sets off the DNA damage response (DDR) in affected cells, which can compromise the effectiveness of anti-cancer drugs due to the engagement of DNA lesion repair mechanisms, ultimately fostering drug resistance [96]. The effectiveness of CDDP is initially observed in a murine model of human lung cancer, but resistance develops with prolonged treatment. Cisplatin-resistant cells show elevated DNA damage repair gene expression and DRC [95]. Notably, human CRC cell lines exhibiting resistance to 5-FU treatment display elevated expression levels of genes associated with DNA repair, including FEN1, FANCG, and RAD23B [37, 97]. Additionally, the administration of 5-FU induces an increase in the expression of p53-target genes involved in DNA damage response and repair processes. The proficient repair of these genomic injuries subsequently results in a reduction of cell cycle arrest and apoptosis within the resistant cell lines compared to their non-resistant counterparts [98].

In patients with triple-negative breast cancer (TNBC), there is a positive correlation between reduced expression of the protein 53BP1, which is involved in the DDR, and an increased likelihood of local recurrence following surgery and radiotherapy. This suggests that 53BP1 may serve as a predictive marker for radio-resistance [99]. Moreover, patients with glioblastoma who undergo ionizing radiation therapy exhibit resistance to treatment as a result of PTEN Y240 phosphorylation, which subsequently enhances DNA repair mechanisms [100]. These studies highlight the significant role of DNA repair capacity (DRC) in cancer therapy resistance. However, while the deregulation of the DDR presents an opportunity to diminish resistance arising from DNA repair, it also introduces the potential for amplifying additional mutations due to genomic instability. The accumulation of mutations may drive a renewed cycle of carcinogenesis. Consequently, the response to DNA damage is a highly complex process within cancer treatment and recurrence, requiring thorough assessment when utilized as a therapeutic approach against cancer.

### Cancer stem cells (CSCs)

CSCs, which exhibit the ability to self-renew and differentiate akin to their normal stem cell counterparts, represent a limited fraction of cancer cells. These cells are postulated to be responsible for the initiation, progression, metastasis, and relapse of tumors [101, 102]. In the context of CRC, the presence of CSCs has been detected, and they are believed to play a substantial role in the development of drug resistance [103]. Various stem cell markers, such as CD133, CD44, and Lgr5, have been employed to identify colorectal cancer CSCs. These specific cells possess the capacity to initiate tumors in vivo and exhibit resilience against conventional chemotherapy and radiation therapies [104].

Numerous mechanisms have been postulated to account for the drug resistance observed in CSCs [105]. Among these proposed mechanisms is the upregulation of ATP-binding cassette (ABC) transporters, which actively extrude drugs from the cells, leading to diminished intracellular drug concentrations. Additionally, CSCs have been discovered to display augmented DNA repair capabilities and resistance to apoptosis, further contributing to their survival in the presence of anti-cancer medications [106]. The importance of inherent cellular characteristics and their interactions with the surrounding tumor microenvironment is increasingly acknowledged as significant factors influencing the state of stemness [107].

The initial observation of the capability of soluble molecules, discharged by the tumor microenvironment, to initiate programs resembling those of CSCs was first established in the context of brain tumors. This establishment involved the critical reliance of the self-regeneration and rapid multiplication of stem cell-like entities on their interaction with endothelial lineage cells [108]. Research investigations in colorectal cancer have demonstrated that cancer-associated fibroblasts (CAFs) secrete hepatocyte growth factor (HGF), osteopontin (also known as secreted phosphoprotein 1; SPP1), and stroma-derived factor 1α (SDF-1α or CXCL12), thereby augmenting cancer cell stemness through the activation of the Wnt pathway [109, 110]. Tumor-associated macrophages also support breast and brain CSCs, further emphasizing the significance of the microenvironment in determining CSC characteristics [111, 112]. Furthermore, the secretion of exosomes and microvesicles by niche cells profoundly impacts CSCs and contributes to the development of drug resistance. For instance, microvesicles generated by breast cancer-associated fibroblasts convey miR-221 to cancer cells, enhancing the population of drug-resistant CD133^hi^ stem cells [113]. Considering this evidence, stemness in cancer can be described as a temporary condition of increased adaptability and resilience, significantly influenced by signals from the microenvironment [114]. This includes interactions with niche components, tumor cells, non-tumor cells, soluble factors, and anticancer treatments.

### Epithelial-mesenchymal transition (EMT)

EMT is a process in which epithelial cells lose their typical characteristics and adopt features of mesenchymal cells. During EMT, epithelial cells lose their polarity and many of their intercellular connections, such as desmosomes, adherens junctions, and tight junctions, causing them to detach from epithelial layers. They then acquire several mesenchymal traits, including increased mobility, invasiveness, resistance to apoptosis, and a significantly higher production of extracellular matrix components [115, 116].

The connection between EMT and drug resistance has been recognized for some time, but the exact mechanisms remain unclear. Advances in CSC research have provided a better understanding of how drug resistance develops in cells undergoing EMT. CSCs are a small subset of cells within the larger tumor population that play a key role in tumor formation [117]. Research has shown that cells undergoing EMT exhibit stem cell-like characteristics, sharing important signaling pathways and drug resistance traits with CSCs [118]. A key mechanism of drug resistance in CSCs is the excessive expulsion of drugs by various cell membrane transporter proteins, particularly those belonging to the ABC transporter family [119, 120]. Cells undergoing EMT overexpress ABC transporters and exhibit a drug resistance phenotype similar to that of CSCs [121]. Saxena et al. demonstrated that the promoters of ABC transporters have multiple binding sites for EMT transcription factors (EMT-TFs). In breast cancer cells, the overexpression of EMT-TFs like Twist, Snail, and FOXC2 enhanced the promoter activity and expression of ABC transporters. These cells exhibited a tenfold increase in resistance to doxorubicin treatment compared to control, non-transfected cells [122].

Signaling pathways that promote the EMT phenotype are known to contribute to drug resistance. For example, TGF-β, a cytokine associated with EMT, has been linked to drug resistance. Studies have shown that TGF-β induces EMT, leading to drug resistance, and that neutralizing TGF-β can restore drug sensitivity [123]. Doxorubicin has been observed to induce TGF-β expression, causing EMT in colon cancer cells, which can be reversed by inhibiting the TGF-β/SMAD4 pathway [124]. Other pathways, such as Wnt and Hedgehog, also contribute to drug resistance by promoting EMT. EMT-TFs like Twist, FOXC2, and FOXM1 promote drug resistance, while FOXF2 suppresses EMT [125, 126]. Additionally, EMT-TFs such as Snail, Slug, and ZEB are associated with drug resistance [127, 128]. The TME also contributes to EMT-driven drug resistance. CAFs, which are part of the tumor stroma, play a crucial role in promoting the proliferative and invasive behavior of cancer cells through cell-cell interactions or extracellular signaling molecules [129]. Fibroblasts aid tumor cells in undergoing EMT through cytokines like IL-6 and transcription factor 21 (TCF21) [130, 131]. Hypoxia is another crucial aspect of the tumor microenvironment that encourages cancer cells to undergo EMT and develop drug resistance. Under hypoxic conditions, the activation of hypoxia-inducible factor-1 alpha (HIF-1α) promotes EMT in hepatocellular carcinoma (HCC) and induces drug resistance by increasing P-glycoprotein (Multidrug Resistance 1; MDR1) expression. Knocking down HIF-1α reverses the EMT phenotype and eliminates the drug-resistant phenotype of HCC under hypoxia, further highlighting the role of hypoxia/HIF-1α in EMT-driven drug resistance [132].

### Drug metabolism

Cancer medications must undergo conversion into their active forms to fully realize their potential in combating tumors. Consequently, if the activity of anti-cancer drugs is diminished or their degradation is amplified, resistance to these medications can develop. For example, the conversion of the anti-neoplastic drug Capecitabine (CAP) to its active form, 5-FU, is mediated by thymine phosphorylase (TP). The inhibition of TP gene transcription, encoded by the extracellular growth factor-1 (ECGF-1) gene, due to methylation, leads to drug resistance to CAP [133, 134]. 5-FU exerts its anticancer effects by inhibiting thymidylate synthase (TS) and incorporating its metabolites into RNA and DNA [135]. The enzymes orotate phosphoribosyl-transferase (OPRT), uridine monophosphate synthetase (UMPS), and UMP kinase (UMPK) are crucial for the transformation of 5-FU into active metabolites that exert anti-cancer effects in tumor cells [136]. Studies have shown that reduced expression of these metabolic enzymes is linked to resistance to 5-FU in patients with CRC, leading to decreased effectiveness of 5-FU treatment [137, 138]. Moreover, TP can convert 5-FU into 5-fluoro-2’-deoxyuridine (FdU), an active form of 5-FU. Research indicates that increased expression of TP is associated with a positive response to 5-FU treatment, suggesting that higher levels of TP can enhance the effectiveness of 5-FU in cancer treatment [139, 140]. Patients exhibiting high expression of these specific enzymes responsible for converting 5-FU into its active form may potentially experience greater benefits from 5-FU treatment [141]. Conversely, most of 5-FU is catabolized to inactive metabolites by dihydropyrimidine dehydrogenase (DPD) in the liver. Upregulation of the DPD gene causes CRC resistance to 5-FU. Interference with DPD activity through methylation of the promoter of the DPYD gene, which encodes the DPD protein, or DPD inhibitors such as 5-ethynyluracil and 5-chloro-2,4-dihydroxypyridine (CDHP) can improve the anti-tumor activity of 5-FU to a greater extent [142–146].

Irinotecan is another drug that inhibits DNA transcription and replication by targeting topoisomerase 1 (TOP-1) [147]. Carboxylesterase 2 (CES2) is a crucial enzyme that converts irinotecan to its more active form, SN-38, a topoisomerase inhibitor [148]. Several studies have shown that upregulation of CES2 expression in CRC increases sensitivity to irinotecan [149–151]. Conversely, enzymes such as cytochrome P450-3A4 (CYP3A4), uridine diphosphate glucuronosyltransferase 1A1 (UGT1A1), and β-glucuronidase cause the inactivation of irinotecan and its active form, SN-38 [152]. Targeting these enzymes may potentially decrease the development of resistance to irinotecan, thereby yielding positive outcomes [153, 154].

### Drug transporters

Membrane transporters play a crucial role in the development of multidrug resistance (MDR) in tumor cells. These transporters facilitate the efflux of chemotherapy drugs or molecular targets from the cell, thereby decreasing the intracellular concentration of antitumor medications [155]. The human genome encompasses over four hundred membrane transporters, which are segregated into two distinct families: ATP-binding cassette (ABC) transporters and solute carrier (SLC) transporters. ABC transporters, including multidrug resistance protein 1 (MDR1; P-gp; ABCB1), breast cancer resistance protein (BCRP; ABCG2), and multidrug resistance-associated protein 1 (MRP1; ABCC1), serve as primary efflux transporters [156, 157]. These membrane proteins (ABC) are responsible for the active transportation of a wide variety of substrates across cell membranes. Overexpression of these transporters in CRC often leads to increased efflux of chemotherapy drugs and subsequent resistance to treatment [158–161]. SLC transporters include organic anion transporters, organic cation transporters, and organic anion transport polypeptides [156].

The initial member of the ABC family, known as P-gp, is found in various normal intestinal cells [162]. P-gp is a prototypical efflux transporter that facilitates multidrug resistance in tumor therapy by actively extruding chemotherapeutic agents from cells. Increased expression of P-gp causes drug resistance, as evidenced by studies showing high P-gp expression in CRC cells resistant to chemotherapy drugs such as 5-FU, doxorubicin, and oxaliplatin [163–165]. BCRP, primarily located in the apical membrane of cancer cells, plays a substantial role in conferring resistance to various anticancer drugs, including topoisomerase inhibitors and tyrosine kinase inhibitors [166–168]. The heightened expression of BCRP in CRC is associated with diminished intracellular accumulation of chemotherapeutic agents, compromising treatment effectiveness [166]. Similarly, MRP1, which functions within the basolateral membrane of cells, is associated with drug resistance in CRC. Its transport capacity, encompassing various substrates such as anthracyclines, taxanes, and antimetabolites, restricts drug accumulation within cancerous cells [169, 170].

In summary, the complex interaction between drug resistance in CRC and ABC transporters underscores the necessity for specific therapeutic interventions. Understanding the molecular mechanisms governing these transporters and devising approaches to regulate their function could potentially facilitate the development of more effective treatment modalities for patients with CRC.​.

## LncRNAs: structure and biogenesis

Long non-coding RNAs (lncRNAs) are the largest class of non-coding RNAs, with lengths ranging from approximately 200 nucleotides to 100 kb [171]. Despite their lack of protein-coding potential, lncRNAs play crucial roles in various cellular processes, including chromatin remodeling, transcriptional and post-transcriptional regulation, mRNA stability, protein function, and cell signaling. Depending on their function, these molecules can be located in different cellular compartments, including the nucleus, cytoplasm, or specific focal regions within the cell [172]. Similar to mRNAs, most lncRNAs consist of exons, introns, 5’ caps, and poly-A tails. However, their sequences and structures are often more complex and less conserved than those of protein-coding genes. Many lncRNAs are transcribed by RNA polymerase II [173, 174]. They tend to be shorter in length compared to mRNAs and typically have fewer exons, although these exons are generally longer [175, 176]. Some lncRNAs can form secondary structures within their sequences, contributing to their functional diversity [177]. Additionally, lncRNAs can adopt tertiary structures, resulting from long-range interactions between distant regions within the RNA molecule [178].

LncRNAs can be categorized into five groups based on their genomic location: sense, antisense, bidirectional, intronic, and intergenic [179]. Sense lncRNAs are positioned within a protein-coding gene and extend across several introns or exons. Antisense lncRNAs are located on the opposite strand of a protein-coding gene. Bidirectional lncRNAs are transcribed from the opposite strand within a vicinity of 1 kb of the promoter of the sense strand. Intronic lncRNAs are situated on the sense strand within an intron of coding genes. Lastly, intergenic lncRNAs are consistently found between two protein-coding genes [180]. In another classification, lncRNAs can be categorized into four groups based on their molecular mechanism [179]; (I) Molecular signals: lncRNAs function as molecular signals, facilitating or inhibiting the transcription factor’s ability to regulate gene expression [181–183]; (II) Decoys: lncRNAs act as decoys or sponges by targeting microRNAs, preventing the miRNAs from executing their function [184, 185]; (III) Guide: lncRNAs have the capacity to attract proteins to specific targets [179, 185]; and Scaffolds: lncRNAs serve as platforms that interact with different proteins involved in specific cellular processes [179]. These molecules play important roles in various biological regulations, such as cell cycle regulation, proliferation, apoptosis, and cell migration [186].

## LncRNAs in CRC

In recent years, there has been growing interest in the crucial role of lncRNAs in diseases and cancers [187–190]. These molecules have the potential to serve as biomarkers for prognosis and treatment response in CRC and other malignancies [188, 191, 192]. lncRNAs can be found in diverse tissues as well as in body fluids such as plasma, serum, urine, and cerebrospinal fluid (CSF) [193, 194]. lncRNAs can function as oncogenes or tumor suppressors, influencing proliferation, apoptosis, invasion, angiogenesis, and metastasis in CRC [184, 185, 195]. Alterations in the expression of lncRNAs regulate the cellular biology of CRC, as compiled in Table 2.

**Table 2:** Different LncRNAs contributed to the pathogenesis of CRC

lncRNA Name	Function	Expression Level	Molecular Mechanism	Model System	Effects on biological processes	Ref.
LINC00668	Carcinogenic role	Upregulated	Regulates the expression of USP 47 by sponging miR-188-5p	Cell lines (HT-29, SW620, SW480, RKO, and LoVo)	Promotes tumorigenesis and progression	ADDIN EN.CITE [190]
NEAT	Carcinogenic role	Upregulated	Regulates the expression of SIRT 1 by sponging mir-34-a	Cell lines (SW620, SW480, HCT116, HT29, CaCo-2, LoVo and Colo205)	Promotes progression	ADDIN EN.CITE [200]
NALT1	Carcinogenic role	Upregulated	Regulates the expression of PEG10 by sponging mir- 574-5p	Cell lines (HT29 and HCT116)	Promotes proliferation, migration, and invasion	ADDIN EN.CITE [201]
MIR4435-2HG	Carcinogenic role	Upregulated	Regulates the expression of YAP 1 by sponging miR-206	Cell lines (HT-29, SW620, LoVo, LS123 and HCT116)	Promotes proliferation and metastasis	[189]
RP11-757G1.5	Carcinogenic role	Upregulated	Regulates the expression of YAP1 by sponging miR-139-5p	Cell lines (HT-29, HCT-116, SW480, SW620, LoVo and Caco-2)	Promotes proliferation and liver, spleen metastasis	ADDIN EN.CITE [202]
MALAT1	Carcinogenic role	Upregulated	Regulates the expression of SOX9 by sponging miR-145	Cell lines (DLD-1 and HT-29)	Promotes proliferation, invasion, and migration	[203]
SNHG5	Carcinogenic role	Upregulated	Regulates the expression of CREB5 by sponging miR-132-3p	Cell lines (SW480 and FHC)	Promotes proliferation, metastasis, and migration	ADDIN EN.CITE [204]
PCAT-1	Carcinogenic role	Upregulated	Regulates cell proliferation by sponging miR-149-5p	Cell lines (SW480, SW620, LOVO and HT29)	Promotes proliferation, invasion, and migration	[205]
TTTY15	Carcinogenic role	Upregulated	Regulates the expression of DVL3 by sponging miR-29a-3p	Cell lines (HCT-116, HT29, SW480, 77 SW620, and DLD-1)	Promotes proliferation, migration, and invasion	[206]
HOXA11-AS	Carcinogenic role	Upregulated	Regulates the expression of PADI2 by sponging 125a-5p	Cell lines (Colo205, HCT116, Lovo, SW620, Caco-2, and SW480)	Promotes metastasis	[207]
MIR17HG	Carcinogenic role	Upregulated	Regulates the expression of NF-κB/RELA by sponging miR-375	Cell lines (HCT15, HCT116, SW480, SW620, HT29, DLD-1, RKO, and LoVo)	Promotes tumorigenesis and metastasis	ADDIN EN.CITE [208]
UICLM	Carcinogenic role	Upregulated	Regulates the expression of ZEB2 by sponging miR-215	Cell lines (SW620, SW480, LoVo, HT-29, HCT116, DLD-1, and RKO)	Promotes liver metastasis	[209]
H19	Carcinogenic role	Upregulated	Interacts hnRNPA2B1 and inducing EMT	Cell lines (HCT116, SW480 and DLD1)	Promotes metastasis	[210]
PCAT1	Carcinogenic role	Upregulated	Regulates the Netrin-1-CD146 complex	Cell lines (HCT116, SW480, and T84)	Promotes liver metastasis	[211]
LOC105369504	Tumor suppressor	Downregulated	Regulates the stability of PSPC1 protein	Cell lines (HCT8, HCT-116, LOVO, and RKO)	Inhibits tumor proliferation and metastasis	[212]
MALAT1	Carcinogenic role	Upregulated	Regulates AKAP-9 expression	Cell lines (SW480)	Promotes proliferation, invasion, and metastasis	ADDIN EN.CITE [186]
MEG3	Tumor suppressor	Downregulated	Regulates p53 and MDM2	Cell lines (HCT-116 and DLD-1)	Inhibits proliferation	[213]
MIAT	Carcinogenic role	Upregulated	Regulates ATM and CHK2	Cell lines (SW116 and SW48)	Promotes Proliferation and Invasion	[214]
SATB2-AS1	Tumor suppressor	Downregulated	Regulates SATB2-AS1	Cell lines (HCT-116, HT-29, SW-620, HCT-8, SW-480, and DLD-1)	Inhibits metastasis	ADDIN EN.CITE [215]
Lnc-LALC	Carcinogenic role	Upregulated	Silences LZTS1 gene	(LoVo, CACO2, DLD1, HT29, HCT116 and SW480)	Promotes liver metastasis	[216]
lncRNA-mAK028845	Carcinogenic role	Upregulated	Regulates KRT37 gene expression	Animal models	Promotes CRC initiation	ADDIN EN.CITE [217]
NALT1	Carcinogenic role	Upregulated	Regulates PEG10 and Ki67 expression	Animal models	Increases tumor volume and growth	[218]
lncRNA-DANCR	Carcinogenic role	Upregulated	-	Animal models	Increases metastasis and tumor growth	[219]
GAS5	Tumor suppressor	Downregulated	-	Animal models	Enhances tumor formation and body weight loss with GAS5 knockdown	[220]

## LncRNAs in colorectal cancer drug resistance

The acquisition of drug resistance traits following treatment is linked to the regulation of gene expression through various non-coding RNAs, including miRNAs and lncRNAs [215, 216]. An illustrative instance of the emergence of 5-FU resistance in CRC is intricately connected with the dysregulation of numerous lncRNAs (Table 3). Among these, urothelial carcinoma-associated 1 (UCA1), stands out as a pivotal player in shaping the response to 5-FU chemotherapy. This lncRNA acts as a sponge for miR-204-5p, thereby intricately modulating cellular processes [217]. The complex interaction between UCA1 and miR-204-5p leads to an indirect increase in the expression of CREB1 (cAMP Response Element-Binding Protein 1), a transcription factor with significant implications for cellular function. This regulatory cascade has been specifically linked to the development of resistance to 5-FU. The elevation of CREB1 levels, orchestrated by the interplay of UCA1 and miR-204-5p, is associated with an unfavorable prognosis, suggesting its involvement in poor overall survival outcomes [217]. Therefore, the multifaceted interactions involving lncRNAs, miRNAs, and their downstream targets, such as CREB1, contribute significantly to the intricate landscape of drug resistance acquisition in CRC.

Another lncRNA that plays a role in 5-FU resistance is GIHCG (Gradually Increased During Hepatocarcinogenesis). This particular lncRNA exhibits heightened expression levels in both CRC tissues and cell lines. The significance of GIHCG extends beyond mere presence, as its overexpression has been correlated with a range of crucial cellular phenomena [218]. The multifaceted role of GIHCG in influencing these critical aspects of cancer progression underscores its potential as a key player in the intricate landscape of drug resistance. There is also supporting evidence indicating that the reduction in expression levels of PVT1 (Plasmacytoma Variant Translocation 1), MALAT1 (Metastasis-Associated Lung Adenocarcinoma Transcript 1), and PCAT-1 (Prostate Cancer-Associated Transcript 1) renders CRC cells more responsive to 5-FU treatment. This downregulation leads to the initiation of both early and late apoptosis by modulating the expression of MDR genes [219–221]. Furthermore, chemoresistance in CRC has been notably associated with the downregulation of two specific elements, namely snaR and SLC25A25-AS1. The downregulation of snaR and SLC25A25-AS1 appears to create a microenvironment within CRC cells that favors resistance to chemotherapeutic agents. This resistance mechanism likely involves intricate molecular pathways and signaling cascades that are yet to be fully elucidated [222, 223].

Certain facets of chemotherapy resistance have been associated with lncRNAs-miRNAs interactions. For instance, the lncRNA ENST00000547547 has been found to enhance sensitivity to 5-FU in CRC cells by competitively inhibiting the miR-31/ABCB9 axis [224]. Conversely, the lncRNA LINC00152 (Cytoskeleton regulator RNA; CY-TOR), through its interaction with miR-139-5p and the subsequent suppression of the NOTCH1 (neurogenic locus notch homolog protein 1) axis, has been implicated in elevating chemoresistance by hindering apoptosis [225].

In the context of CRC treatment with oxaliplatin, a spectrum of lncRNAs emerges as influential players in promoting apoptosis and inducing cytotoxicity. This group includes GIHCG [218], LIN00152 [226], MALAT1 [227], H19 [228], and MEG3 (Maternally Expressed Gene 3) [229, 230]. These lncRNAs orchestrate their effects through various mechanisms, predominantly by forming intricate axes with miRNAs that target pivotal genes involved in regulating cellular death processes. Conversely, when examining cisplatin/CDDP resistance in CRC, the central mediators are identified as HOTAIR (HOX Transcript Antisense Intergenic RNA) and PVT1. These specific lncRNAs influence apoptotic pathways, manipulate the expression levels of miR-203a-3p, and affect the activity of the Wnt/β-catenin signaling pathway. This intricate network of interactions underscores the multifaceted nature of CRC resistance to cisplatin, highlighting the need for a comprehensive understanding of these molecular pathways [231, 232]. Notably, H19 contributes to drug resistance during methotrexate treatment through the Wnt/β-catenin signaling pathway [233]. In the case of TUG1 (Taurine Up-Regulated Gene 1), resistance is attributed to the modulation of the CPBE2 (Cytoplasmic Polyadenylation Element Binding Protein 2) gene following the inhibition of miR-186 [234]. Lastly, resistance to doxorubicin is predominantly influenced by the XIST (X-Inactive Specific Transcript)/miR-124/SGK1 (Serum/Glucocorticoid Regulated Kinase 1) axis, which actively promotes chemoresistance in CRC cells [235].

Assessing the expression profiles of lncRNAs holds significant importance, as it enables the identification of new biomarkers indicative of resistance in CRC. These biomarkers can serve as promising therapeutic targets, leveraging their biological characteristics to enhance the effectiveness of chemotherapy in CRC patients. Through such insights, there exists potential to elevate the efficacy of treatment strategies, thereby advancing the management of CRC resistance and improving patient outcomes.

**Table 3:** LncRNAs in CRC drug resistance

lncRNAs	Function	Expression Level	CRC cell lines	Mechanism of Resistance	Ref.
GIHCG	Carcinogenic role	Upregulated	SW620, HT29, HCT8, HCT116, LoVo, SW480, DLD1, HCoEpic	Potential target in 5-FU and Oxaliplatin resistance mechanisms.	[223]
Mir100HG	Carcinogenic role	Upregulated	HCA-7	Coordinately MIR100HG, miR-100 and miR-125b overexpression drives Cetuximab resistance by targeting five negative regulators of Wnt signaling which have a potential clinical relevant interaction with EGFR.	[241]
UCA1	Carcinogenic role	Upregulated	HEK-293T, HCT8, HCT116, HT29, LoVo and SW480, Caco2	UCA1 can decrease the sensitivity of CRC cells to 5-FU by sponging miR-204-5p resulting in attenuating apoptosis. Moreover, UCA1 expression levels are increased in Cetuximab resistant cells and can be transferred to sensitive cells through exosomes increasing resistant cells number. Increased expression of UCA1 leads to increased expression of important drug resistance genes such as MDR1, ABCB1 and FOXM1.	ADDIN EN.CITE [222, 242, 243]
LIN00152	Carcinogenic role	Upregulated	SW480, Caco2, SW620, HT29, HCT8, HCT116, LOVO	LIN00152 leads to resistance to Oxa and 5-FU by absorbing miR-193a-3p through ERBB4 modulation, which activates the AKT signaling pathway that enables cell survival and chemoresistance. miR-193a-3p also controls NOTCH1 to regulate CRC growth, metastasis, stemness, and chemoresistance.	[230, 231]
HOTAIR	Carcinogenic role	Upregulated	CCD18Co, HCT15, CoLo205, HT29, DLD-1, SW620	HOTAIR may control the advancement of chemoresistance in CRC by targeting miR-203a-3p and the Wnt/b-catenin signaling pathway.	[237]
PCAT-1	Carcinogenic role	Upregulated	Caco-2, HT29	PCAT-1 controls invasiveness and 5-FU resistance in CRC cells. PCAT-1 may promote CRC cell invasion through c-Myc expression modulation.	[224]
PVT1	Carcinogenic role	Upregulated	HCT-8, HCT-116	PVT1 predicts resistance to 5-FU in CRC tissues and cells by blocking cell death and increasing the levels of MRP1, P-gp, mTOR, and Bcl-2.	[225]
MALAT1	Carcinogenic role	Upregulated	HCT-116, SW480, COLO205, LOVO, SW620, HT29	Overexpression of MALAT1 increases resistance to chemotherapy in cells that are resistant to 5-FU by enhancing the expression of genes that are involved in multidrug resistance, including MDR1, MRP1, BCRP, and ABC. Additionally, MALAT1 modulates the EZH2 pathway in cells that are resistant to Oxa.	[226, 232]
H19	Carcinogenic role	Upregulated	HT-29, HCT116, SW480	H19 activates Wnt/b-catenin signaling, making it a potential target for MTX resistant CRC. CAFs transfer exosomal H19 to CRC cells, promoting stemness and Oxa chemoresistance by sponging miR-141.	[233, 238]
SLC25A25-AS1	Tumor suppressor	Downregulated	HCT-116, HT-29	SLC25A25-AS1 plays a crucial part in CRC cells by enhancing the effectiveness of chemotherapy drugs through the modulation of the Erk and p38 pathways. Therefore, it was found that SLC25A25-AS1 has a tumor suppressive role in CRC.	[227]
snaR	Tumor suppressor	Downregulated	SNU-C4, SNU-C5, HCT116	snaR negatively regulates CRC cell growth and is involved in the development of 5-FU resistance. However, the specific roles of snaR are still unclear.	[228]
ENST00000547547	Tumor suppressor	Downregulated	HCT116, LoVo	ENST00000547547 decreased the resistance to 5-FU by competitively acting as a sponge for miR-31, which in turn targets ABCB9, a gene associated with apoptosis induced by chemotherapy. This implies that the lncRNA ENST00000547547 could serve as a favorable prognostic factor for 5-FU-based chemotherapy.	[244]
TUG1	Carcinogenic role	Upregulated	HT29, HCT8	TUG1 facilitates resistance to methotrexate (MTX) in colorectal cancer by acting as a sponge for miR-186. This interaction inhibits the suppressive effect of miR-186 on CPEB2, leading to an increase in the protein levels of CPEB2. Notably, elevated levels of CPEB2 are implicated in both tumorigenesis and chemoresistance, underscoring the significant role played by TUG1 in these processes.	[239]
MEG3	Tumor suppressor	Downregulated	SW480, HT29, HCT116,	MEG3 actively enhances chemosensitivity to oxaliplatin (Oxa) by instigating cytotoxicity in colorectal cancer (CRC) cells, thereby promoting apoptosis. Additionally, MEG3 operates as a sponge for miR-141, counteracting its inhibitory effects on the tumor suppressor gene PDCD4.	[234, 235]

### LncRNAs targeting apoptosis

Evidence has documented the pro-apoptotic involvement of lncRNAs in CRC. lncRNAs interact with various molecules both upstream and downstream in apoptotic pathways [239–241]. Oncogenic lncRNAs exhibit increased expression, enhancing pathways that inhibit apoptotic processes [242, 243]. The majority of identified lncRNAs regulate TRAIL (tumor necrosis factor-related apoptosis-inducing ligand)-mediated apoptosis by either promoting or inhibiting the expression of Bcl-2, contributing to heightened tumor growth, increased metastatic potential, and enhanced resistance to chemotherapy. In CRC, the expression of long non-coding RNA small Cajal body-associated RNA2 (scaRNA2) is notably increased, which is linked to larger tumor size, chemoresistance, and metastasis. Evidence suggests that scaRNA2 contributes to tumor proliferation by fostering the expression of Bcl-2 and EGFR [244].

In a study, the tumor-suppressive properties of miR-342 were investigated, revealing that its expression is suppressed by epigenetic modification [245]. The relationship between scaRNA2 and miR-342 expression is inversely correlated. Research by Lai et al. demonstrated that miR-342 promotes apoptosis by inhibiting Bcl-2 expression and inducing Bax expression. Conversely, decreased miR-342 expression leads to resistance to chemotherapy in CRC cells by reinforcing the anti-apoptotic pathway [246]. Similarly, lncRNA PCAT-1 exhibits high expression in CRC tissues and corresponding cell lines. Increased expression of PCAT-1 is positively correlated with CRC tumor progression, metastasis, and poor overall survival [247]. In a study, PCAT-1 expression was suppressed in Caco-2 and HT-29 cells using shRNA2-PCAT-1 complementary treatment. The decrease in PCAT-1 expression led to reduced levels of cyclin D1 and cyclin E, resulting in cell cycle arrest and inhibition of cell growth. The rate of apoptosis increased significantly after treatment with shRNA2-PCAT-1, causing apoptosis by increasing the expression of Bax and cleaving PARP (Poly (ADP-ribose) polymerase) and caspase 3, while suppressing the expression of Bcl-2 [248].

lncRNA Lung Cancer-Associated Transcript 1 (LUCAT1) plays a role in regulating the apoptotic pathway by suppressing the expression of PARP-1. In CRC, LUCAT1 is overexpressed and is closely associated with an unfavorable prognosis and more advanced tumor stages. Research findings indicate that the suppression of LUCAT1 reduces cell viability, induces cell growth arrest, and increases the stability of the p53 protein [249]. Additionally, lncRNA Zinc Finger Antisense 1 (ZFAS1) has been shown to cause tumor progression by destabilizing p53. Inhibition of ZFAS1 results in a diminished capacity for colony formation, induces cell cycle arrest, impedes cell progression, and promotes apoptosis through increased PARP cleavage, upregulation of p53 expression, and reduced cyclin B1 levels [250].

Knockdown of LINC00908, an oncogenic lncRNA in CRC tissues and cells, triggers intrinsic apoptosis by enhancing cleavage activity, resulting in increased levels of caspase-9 and caspase-3 [251]. Zhang and colleagues [252] investigated the correlation between the expression levels of the lncRNA XIST and the clinicopathological characteristics of CRC. Their findings revealed significant upregulation of XIST in CRC, linked to enhanced cell proliferation and unfavorable patient survival. XIST functions as a miRNA sponge, influencing cell proliferation, metastasis, and apoptosis in CRC. Experimental silencing of XIST reduced cell proliferation and migration while triggering apoptosis.

The lncRNA FOG family member 2 antisense RNA 1 (ZFPM2-AS1) is highly expressed in CRC and is linked to cell proliferation, migration, and invasion. ZFPM2-AS1 acts as a competing endogenous RNA (ceRNA), binding with miR-137 and upregulating the expression of tripartite motif-containing 24 (TRIM24), a downstream target of miR-137 [253]. Upregulation of TRIM24 is associated with increased proliferation and inhibition of apoptosis in CRC cells. Knockdown of TRIM24 results in decreased Bcl-2 expression and increased expression of caspase 3 and PARP, proteins involved in apoptosis regulation [254, 255].

Another lncRNA, LINC02474, acts as an oncogene. High levels of LINC02474 are associated with increased invasion and decreased apoptosis. LINC02474 is implicated in the suppression of apoptosis in CRC through the downregulation of Granzyme B (GZMB) expression. GZMB, a serine protease enzyme secreted by cytotoxic T lymphocytes and natural killer cells, plays a pivotal role in apoptosis by inducing the hydrolytic cleavage of caspase-3 and Bid [256, 257].

The initiation of the extrinsic apoptotic pathway in CRC is triggered by ligands such as TRAIL, FasL, or tumor necrosis factor-alpha (TNF-α) interacting with their respective death receptors. The lncRNA ST3GAL6 Antisense RNA 1 (ST3GAL6-AS1; ST3 beta-galactoside alpha-2,3-sialyltransferase 6 antisense RNA) indirectly influences this pathway by suppressing PI3K/Akt signaling and promoting the nuclear translocation of Forkhead box protein O1 (FOXO-1) [258, 259]. This process aids apoptosis by enabling the transcription of FasL, Bim, BAD, and TRAIL by FOXO-1 [260]. Overexpression of ST3GAL6-AS1 has been found to correlate negatively with tumor stage, size, and metastasis in patients with CRC [259].

The lncRNA Growth Arrest-Specific 5 (GAS5) regulates apoptosis by controlling FOXO3a expression. Similar to FOXO-1, FOXO3a induces apoptosis via FasL and TRAIL expression. GAS5 is downregulated in CRC tissues and certain cell lines, correlating with lymph node metastasis and advanced cancer stages. Overexpression of GAS5, achieved by transfecting pcDNA3.1-GAS5 plasmids into CRC cell lines, reduces cell proliferation and increases apoptosis. GAS5 promotes apoptosis by inhibiting miR-182-5p and upregulating FOXO3a expression [261, 262].

The lncRNA LINC00152 (CYTOR) exhibits diminished expression in colon cancer cell lines. Transient expression of LINC00152, achieved via lentivirus-based techniques, leads to decreased cell viability and fosters apoptosis by upregulating FasL and downregulating Bcl-2 [263]. Conversely, other studies have reported elevated expression of LINC00152 in CRC tumor tissues, which is associated with growth-promoting functions, poor prognosis, and increased cancer cell migration, invasiveness, and chemoresistance [225, 264]. Another lncRNA, LINC00460, Long Intergenic Non-Protein Coding RNA 460, identified through microarray profile analysis of CRC, displays high expression levels that correlate with tumor proliferation in CRC cell lines. Its overexpression is linked with advanced tumor stages, lymph node metastasis, and reduced survival. Knockdown of LINC00460 triggers the activation of caspase 3 and downregulates the expression of TRAIL, caspase-9, Bax, and Bcl-2, suggesting that LINC00460 inhibits TRAIL-mediated apoptosis [265].

The p38 pathway plays a pivotal role in TRAIL-induced apoptosis, mediating the transcription of TRAIL and death receptor 5, DR5 [266, 267]. However, in certain cancer cell lines, the role of p38 is associated with cell growth and proliferation. The lncRNA SLC25A25-AS1, which exhibits lower expression in CRC tumor tissues and several CRC cell lines, regulates the phosphorylation activation of p38 [222, 268]. Its under-expression leads to chemoresistance. Induced expression of SLC25A25-AS1 inhibits p38 activation and correlates with suppressed cell proliferation and diminished colony-forming ability [222].

The lncRNA LINC00958, Long Intergenic Non-Protein Coding RNA 958, has been shown to be overexpressed in colorectal cancer. This overexpression is associated with reduced apoptosis, increased proliferation, and suppressed radiosensitivity. It acts as a miR-422a sponge, preventing miR-422a from inhibiting MAPK1 expression, a key player in apoptosis [269, 270]. Additionally, recent studies have shown that CAF-derived exosomal lncRNA FAL1, Focally amplified lncRNA on chromosome 1, increases chemoresistance to oxaliplatin by regulating autophagy in CRC [271].

Given the role of certain dysregulated lncRNAs in inhibiting apoptosis, strategies to attenuate their expression could potentially serve as effective therapeutic approaches for CRC.

### LncRNAs targeting DNA damage response and repair

A recently discovered role of lncRNAs is their involvement in the regulation of the DNA damage response (DDR) [272, 273]. They achieve this by modulating various DDR signaling pathways, including the critical ATM (ataxia-telangiectasia, mutated) and ATR (ATM and Rad3-related) pathways, as well as the p53 pathway. For example, lncRNA DDSR1 (DNA damage-sensitive RNA1) is induced by DNA damage. DDSR1 induction is triggered by several DNA double-strand break (DSB) factors in an ATM-NF-κB pathway-dependent manner [274]. The depletion of DDSR1 has been shown to hinder cell proliferation and DDR signaling, and it diminishes the capacity for DNA repair through homologous recombination (HR). This suggests that DDSR1 plays a crucial role in maintaining cellular health and genomic stability. The loss of DDSR1 could therefore have significant implications for cellular function and response to DNA damage [274].

The lncRNA LINC01021 has been shown to directly interact with the p53 protein, leading to the binding of p53 to the MER61C Long Terminal Repeat (LTR) located on its promoter region. This interaction results in the upregulation of LINC01021 in a p53-dependent manner. Importantly, this upregulation has been linked to the modulation of chemotherapy resistance and the DDR in CRC cells, suggesting a potential role for LINC01021 in influencing therapeutic response and DDR in CRC [275]. Additionally, the lncRNA BRAF-activated noncoding RNA (BANCR) has been found to be upregulated in CRC, linked to both tumorigenesis and chemoresistance. It is hypothesized that BANCR acts as a sponge for miR-203, which in turn enhances DNA repair responses by upregulating the expression of CSE1L in CRC [276]. The CSE1L gene, also known as human chromosomal segregation 1-like, plays a crucial role in the cell nucleus and is involved in processes such as cell proliferation, migration, and apoptosis. Overexpression of CSE1L has been associated with tumor development and carcinogenesis, further highlighting the complex interplay between lncRNAs and other cellular components in cancer [277]. The lncRNA Colorectal Neoplasia Differentially Expressed (CRNDE) has been identified as a novel lncRNA overexpressed in various types of cancer, particularly in CRC. Overexpression of CRNDE enhances cell viability, metastasis, resistance to the chemotherapy drug oxaliplatin (OXA), and DNA repair. This is achieved through the endogenous sponging of miR-136, leading to the upregulation of E2F1 transcription factor. This highlights the multifaceted role of lncRNAs in cancer progression and treatment response [278].

The lncRNA known as p53-Induced Noncoding RNA (PINCR) has been shown to be overexpressed in response to DNA damage. This overexpression regulates genes targeted by p53, which are involved in G1 arrest and apoptosis. PINCR is directly induced by p53 and exerts its role by sponging the RNA-binding protein Matrin3. This interaction is particularly significant in response to DNA damage in CRC, further emphasizing the complex roles of lncRNAs in cellular responses to DNA damage [279]. Research has shown that the intracellular reduction of Matrin3 results in increased DNA damage and sensitivity to radiotherapy [280].

The non-homologous end-joining (NHEJ) repair pathway, regulated by lnc-RI (Long noncoding RNA Regulator of DNA damage repair and Radiosensitivity), influences CRC cell growth and radiosensitivity. lnc-RI achieves this by modulating LIG4 (DNA ligase IV) expression via the lnc-RI/miR-4727-5p/LIG4 axis, thereby affecting the efficiency of NHEJ repair in DNA damage repair [281]. It has been reported that lncRNA MALAT1, which is highly expressed in CRC cells, influences CRC radiosensitivity by modulating DNA damage repair. Furthermore, the knockdown of lncRNA MALAT1 can diminish CRC radioresistance by adjusting DNA damage repair via the YAP1/AKT axis [282].

In summary, the data presented underscores the significant role of lncRNAs in the tumorigenesis of CRC, particularly through their influence on DNA damage response mechanisms and chemotherapy resistance.

### LncRNAs targeting drug metabolism and drug transporters

Modifications in drug metabolism constitute one of the key and most extensively researched mechanisms contributing to drug resistance. The processes involved in drug metabolism and disposition can be broadly classified into three categories: Phase I, Phase II, and Phase III. Each phase plays a distinct role in the metabolism and elimination of drugs, thereby influencing their efficacy and the development of drug resistance [283]. lncRNAs possess the capability to modulate specific Phase I enzymes, thereby influencing drug resistance in neoplastic cells. For instance, in colorectal cancer, the heightened expression of lncRNA H19 corresponds with an elevation in intracellular aldehyde dehydrogenase (ALDH) activity. Notably, lncRNA H19 initiates the activation of the β-catenin pathway through the sequestration of miR-141. This molecular interaction contributes to tumor progression and the development of chemoresistance in CRC [228]. lncRNAs have also been demonstrated to influence the regulation of distinct Phase II enzymes [284]. The expression of the lncRNA HOTAIR exhibits a positive correlation with both the levels of chondroitin sulfotransferase (CHST15) protein in primary tumors and the quantity of metastatic tumor lesions [285].

Recent investigations have revealed that certain lncRNAs can influence various ABC transporters, leading to the development of drug resistance. Moreover, there is a noteworthy elevation in the levels of lncRNA very low-density lipoprotein receptor (VLDLR) in HCC. The suppression of linc-VLDLR results in a significant reduction in HCC proliferation and the expression of BCRP/ABCG2. Conversely, the heightened expression of BCRP/ABCG2 diminishes the impact of lncRNA VLDLR knockdown on sorafenib-induced cell death in HepG2 cells [286]. The expression of the lncRNA PVT1 is observed to be high in cisplatin-resistant gastric cancer cells. Furthermore, PVT1 up-regulation resulted in higher expression levels of MDR1, MRP, mTOR, and HIF-1α [287].

Numerous studies have highlighted the role of lncRNAs in regulating the expression of drug transporters via microRNA sponging. A prime example is the lncRNA XIST, which has been demonstrated to positively regulate Serum/Glucocorticoid Regulated Kinase 1 (SGK1), a known positive regulator of transporters. This regulation occurs through the direct sponging of the SGK1-targeting microRNA, miR-124. This interaction has been linked to resistance to the chemotherapy drug doxorubicin in both colorectal cancer tissues and cell lines [235].

lncRNAs have been recognized as potential modulators of drug transporter proteins, particularly within the realm of cancer chemotherapy. These lncRNAs may play a crucial role in mediating the efficacy and response to therapeutic agents [284]. For instance, the lncRNA MALAT1 can modulate the expression of efflux transporters, namely MRP1 and MDR1, through the activation of the transcriptional factor STAT3. In A549 lung cancer cells resistant to cisplatin, elevated MALAT1 expression fosters an increase in MRP1 and MDR1 expression, leading to reduced cisplatin sensitivity. Niclosamide, a specific inhibitor of STAT3, can negate the upregulation of MRP1 and MDR1 induced by MALAT1, highlighting the potential of targeting STAT3 to modulate drug resistance mediated by lncRNAs in cancer therapy [288].

ANRIL, Antisense Non-coding RNA in the INK4 Locus, is implicated in the regulation of MDR1 and MRP1. It is highly expressed in cells resistant to cisplatin and in 5-FU-resistant gastric cancer tissues and cells. Knockdown of ANRIL results in decreased cell proliferation and expression of MDR1 and MRP1. A robust correlation between ANRIL and MDR1/MRP1 expression has been observed in gastric cancer tissues from patients. Further experimental evidence demonstrated that ANRIL knockdown reverses resistance to cisplatin and 5-FU in gastric cancer cells [289]. MRUL (MDR-related and upregulated lncRNA) is a lncRNA that upregulates ABCB1 and induces multidrug resistance. Knockdown of MRUL increases apoptosis and decreases doxorubicin flow in a gastric cancer cell line [290]. Numerous studies have highlighted the role of lncRNAs in regulating the expression of drug transporters via microRNA sponging. A prime example is the lncRNA XIST, which has been demonstrated to positively regulate Serum/Glucocorticoid Regulated Kinase 1 (SGK1), a known positive regulator of transporters. This regulation occurs through the direct sponging of the SGK1-targeting microRNA, miR-124. This interaction has been linked to resistance to the chemotherapy drug doxorubicin in both CRC cells and tissues [235]. The lncRNA KCNQ1OT1 has been reported to regulate resistance to the chemotherapy drug oxaliplatin through the miR-7-5p/ABCC1 pathway. By modulating this pathway, KCNQ1OT1 can influence the sensitivity of cancer cells to oxaliplatin, thereby affecting the efficacy of chemotherapy [291]. Additionally, LINC00518 can act as a molecular sponge for MRP1 (ABCC1), specifically targeting miR-199a. This interaction leads to increased MRP1 expression and subsequently enhanced chemoresistance. Knockdown of LINC00518 has been observed to increase chemosensitivity to adriamycin, vincristine, and paclitaxel in a chemotherapy-resistant breast cancer cell line [292]. Furthermore, the lncRNA bladder cancer-associated transcript-1 (BLACAT1) has been reported to increase resistance to the chemotherapy drug oxaliplatin by promoting the expression of the ABCB1 protein through the sponging of miR-361 [293].

### LncRNAs targeting CSCs

CSCs represent a small subset of cancer cells with self-renewal and differentiation capabilities, contributing to tumor heterogeneity, metastasis, and drug resistance. In CRC, lncRNAs have been shown to regulate CSCs. Lnc34a is highly expressed in colon CSCs and can initiate asymmetric division, thereby promoting the self-renewal of colon CSCs [294]. The overexpression of lncRNA TUG1 in CRC stem cells is associated with increased resistance to oxaliplatin. Knockdown of lncRNA TUG1 reduces this resistance [295]. LncRNA PTPRG-AS1 has been shown to maintain stem cell-like characteristics and promote resistance to oxaliplatin in CRC by regulating the miR-665 and STAT3 axis [296]. Additionally, lncRNA RBM5-AS1 is overexpressed in colon cancer stem-like cells, promoting cell growth and survival through the activation of the WNT signaling pathway via interaction with β-catenin [297]. The overexpression of lncRNA CCAT2 has been linked to the enhancement of various molecular markers associated with CSCs in colon cancer cells, suggesting its significant role in the progression and maintenance of CSCs [298]. LncRNA LINC01567, also known as LOCCS, is highly expressed in CSCs marked by CD133^+^, CD166^+^, and CD44^+^. Inhibition of LINC01567 effectively reduces the proliferation, migration, invasion, and tumor xenograft formation of colon CSCs by acting as a molecular decoy for miR-93 [299]. Additionally, lncRNA linc01106 has been implicated in promoting colon cancer cell proliferation, migration, and the maintenance of stem cell-like properties through the regulation of the Gli family of transcription factors, partially mediated by the sequestration of miR-449b-5p [300]. lncRNA cCSC1 is highly expressed in colorectal cancer and CRCSCs, leading to increased resistance to 5-FU. Manipulating the expression of lncRNA cCSC1, either through silencing or upregulation, significantly inhibits the self-renewal capacity of CRCSCs and reduces their resistance to 5-FU [301].

### EMT-Related LncRNAs

The process of EMT within the context of CRC is orchestrated through an intricate network of signaling pathways and transcriptional regulation. Central to this process are pivotal signaling cascades including the TGF-β, the Wnt/β-catenin, and the PI3K/AKT pathways. These pathways collectively engage in the activation of transcription factors known to induce EMT, notably Snail, Slug, Twist, and ZEB1/2. These transcription factors play a critical role in modulating gene expression, leading to the downregulation of epithelial markers such as E-cadherin, thereby diminishing cell adhesion and polarity. Concurrently, there is an upregulation of mesenchymal markers like N-cadherin and vimentin, which are instrumental in enhancing cell motility and invasiveness. This molecular reprogramming underpins the transition from a static epithelial phenotype to a more dynamic and invasive mesenchymal state, significantly contributing to the metastatic potential of CRC [302–304]. LncRNAs contribute to both CRC drug resistance and CRC EMT, for instance, LncRNA H19 was found to be upregulated in CRC cells treated with hypoxia or oxaliplatin, contributing to drug resistance both in vitro and in vivo. H19 functions as a ceRNA for miR-675-3p, thereby modulating EMT. Notably, miR-675-3p mimics were able to mitigate the effects of H19 deficiency in CRC cells with hypoxia-induced chemoresistance [305]. H19 acts as a ceRNA that sponges miR-194, a microRNA that normally suppresses autophagy and stemness. By inhibiting miR-194, H19 promotes autophagy and enhances the CSC-like properties of CRC cells, rendering them more resistant to chemotherapy [306, 307].

MALAT1 contributes to oxaliplatin resistance and EMT in CRC. MALAT1 knockdown enhanced E-cadherin expression and suppressed EMT, improving oxaliplatin sensitivity. Mechanistically, MALAT1 interacted with EZH2, which repressed E-cadherin expression, promoting EMT and resistance. Targeted inhibition of MALAT1 or EZH2 reversed oxaliplatin-induced EMT and chemoresistance. MALAT1 also interacted with miR-218, highlighting its prognostic significance in CRC patients receiving FOLFOX therapy [227]. LINC02257 is significantly overexpressed in CRC, associated with poor survival rates and advanced tumor stages. It enhances CRC cell metastasis and proliferation by acting as a ceRNA, sequestering miR-1273 g-3p, which upregulates SERPINE1 expression. Additionally, LINC02257 interacts with Y-box binding protein 1 (YB1), promoting its phosphorylation and nuclear translocation, which activates oncogenic gene transcription [308]. Moreover, lncRNA FEZF1-AS1 (FEZ family zinc finger 1 antisense RNA 1) is highly expressed in CRC and linked to poor patient prognosis. FEZF1-AS1 promotes growth, proliferation, and EMT in CRC cells (HCT116) via the P53 signaling pathway. Knockdown of FEZF1-AS1 induces cell cycle arrest, apoptosis, and suppression of EMT, reducing CRC cell viability, migration, and invasion [309]. Conversely, LINC01550 was downregulated in CRC tissues, correlating with advanced stage, metastasis, and poorer survival. It was an independent risk factor for CRC prognosis. Low LINC01550 expression was linked to higher APC and TP53 mutation rates, while high expression improved sensitivity to several chemotherapy agents such as 5-fluorouracil, irinotecan, trametinib, gemcitabine, rapamycin, and XAV939. Overexpression of LINC01550 inhibited proliferation, migration, invasion, and EMT in HCT-116 and HT-29 cells, while promoting apoptosis by suppressing Wnt/β-catenin signaling [310]. LncRNA Neighboring Enhancer of FOXA2 (lncRNA-NEF) is a tumor suppressor and downregulated in CRC. Functional studies revealed that lncRNA-NEF suppresses oxaliplatin resistance and EMT in vitro and inhibits metastasis in vivo. lncRNA-NEF enhances DOK1 (a MEK/ERK signaling inhibitor) expression by disrupting DNA methyltransferase (DNMT)-mediated methylation, leading to MEK/ERK signaling inactivation [311].

lncRNA NORAD is responsible for hypoxia-induced chemoresistance and vasculogenic mimicry in CRC by acting as a sponge for miR-495-3p. Under hypoxic conditions, CRC cells show an increased capacity for VM formation and resistance to 5-FU, which was associated with elevated NORAD expression. Silencing NORAD inhibits hypoxia-induced VM formation and the expression of the VM marker VE-cadherin. Furthermore, NORAD knockdown reduces CRC cell resistance to 5-FU by lowering cell viability and promoting apoptosis. The loss of NORAD decreased hypoxia-induced HIF-1α expression and subsequent EMT by upregulating E-cadherin and downregulating N-cadherin expression [312]. lncRNA-ENST00000543604 (lncRNA 604) contributes to the progression of CRC through two distinct signaling axes: the lncRNA 604/miRNA 564/AEG-1/EMT pathway and the lncRNA 604/ZNF326/EMT pathway. LncRNA 604 also increases CRC cell chemoresistance by inducing the expression of AEG-1, NF-κB, and ERCC1 [313]. HOTAIR promotes EMT by interacting with the Polycomb Repressive Complex 2 (PRC2) and modifying chromatin to repress E-cadherin expression, facilitating a mesenchymal phenotype and enhancing chemoresistance in CRC [314, 315]. ZEB2-AS1 promotes the expression of the ZEB2 transcription factor, a key EMT regulator, which represses E-cadherin and induces resistance to chemotherapeutics in CRC cells [316]. LncRNA NEAT1, in addition to activating Wnt/β-catenin signaling, promotes the self-renewal and stemness of CRC cells, contributing to both EMT and drug resistance. The dual role of NEAT1 in regulating EMT and CSC-like properties underscores the complex mechanisms by which lncRNAs drive chemoresistance [317–319].

LncRNA CCAT1 (Colon cancer-associated transcript 1) plays a crucial role in CRC by activating the Wnt/β-catenin signaling pathway, enhancing cell proliferation and survival [320]. This activation is linked to resistance against chemotherapeutic agents such as 5-FU and oxaliplatin [321, 322]. By driving Wnt-induced EMT, CCAT1 increases the mesenchymal properties of CRC cells, enabling them to evade the cytotoxic effects of these treatments [323, 324]. Similarly, the PI3K/AKT pathway, another key regulator of EMT, is associated with increased cell survival, proliferation, and drug resistance when activated. LncRNA SNHG1 (Small nucleolar RNA host gene 1) promotes resistance to oxaliplatin in CRC by activating the PI3K/Akt pathway, which enhances EMT, suppresses apoptosis, and boosts cell survival. Through the regulation of this pathway, SNHG1 helps CRC cells resist the DNA damage caused by chemotherapy, contributing to drug resistance [325, 326]. Given the critical role of lncRNAs in regulating EMT and driving drug resistance in CRC, targeting this crosstalk presents a promising therapeutic strategy.

## Conclusions

lncRNAs play a crucial role in CRC drug resistance, with specific lncRNAs such as HOTAIR, MALAT1, and CCAT1 implicated in processes like apoptosis, DNA damage repair, CSC regulation, and drug metabolism. These molecular mechanisms provide key insights into the pathways by which resistance to CRC treatment develops and potential points for therapeutic intervention.

Targeting lncRNAs involved in essential pathways offers promising new strategies to overcome drug resistance in CRC. The identification of lncRNAs specific to critical functions, such as MALAT1 in apoptosis and CCAT1 in DNA damage response, highlights their utility as potential therapeutic targets. Additionally, a deeper understanding of lncRNA structure and biogenesis contributes to more targeted approaches that could enhance therapeutic precision and efficacy.

As research advances, incorporating lncRNA-based strategies into the broader scope of CRC treatment could pave the way for more personalized therapies. Ultimately, these findings hold significant promise for improving patient outcomes and addressing the challenge of drug resistance in colorectal cancer treatment.

## Supplementary Information

Supplementary Material 1

## Data Availability

No datasets were generated or analysed during the current study.
