# Supplementary material for: The lncrnas: innovative multifunctional players of drug resistance in colorectal cancer
Source: Cancer Cell Int. 2025 Nov 23;25:442. doi: 10.1186/s12935-025-03951-3 (PMC12751451; doi:10.1186/s12935-025-03951-3)
Supplement: Supplementary file 1 — Supplementary Material 1 [file 12935_2025_3951_MOESM1_ESM.pdf]

SPRINGER NATURE

Change of authorship request form - Journals (pre-acceptance)

Important information. Please read.

- This form should be used by authors to request any change in authorship (adding/deleting authors) including changes in corresponding authors. This form should not be used for name changes. Please fully complete all sections. Use black ink and block capitals and provide each author's full name with the given name first followed by the family name.

- By signing this declaration, all authors guarantee that the order of the authors are in accordance with their scientific contribution, if applicable as different conventions apply per discipline, and that only authors have been added who made a meaningful contribution to the work.

- Please note, in author collaborations where there is formal agreement for representing the collaboration, it is sufficient for the representative or legal guarantor (usually the corresponding author) to complete and sign the Authorship Change Form on behalf of all authors, **next to the added/removed author(s)**. **(Complete Section 3, followed by Section 6.)**

  In author collaborations where there is no formal agreement for representing the collaboration and **there are more than 10 authors**, one may sign for all, provided the signer appends correspondence that attests that each of the authors have agreed to the change **and the added/removed authors sign the form**. **(Complete Section 3, followed by Section 6.)**

- Please note, we cannot investigate or mediate any authorship disputes. If you are unable to obtain agreement from all authors (including those who you wish to be removed) you must refer the matter to your institution(s) for investigation. Please inform us if you need to do this.

- If you are not able to return a fully completed form within **30 days** of the date that it was sent to the author requesting the change, we may have to withdraw your manuscript. We cannot publish manuscripts where authorship has not been agreed by all authors (including those who have been removed).

- Incomplete forms will be rejected.

- Please return/upload this form, fully completed, to the Journals Editorial Office. The Journal and/or Publisher will consider the information you have provided to decide whether to approve the proposed change in authorship. We may decide to contact your institution for more information or undertake a further investigation, if appropriate, before making a final decision.

SPRINGER NATURE

Change of authorship request form - Journals (pre-acceptance)

Section 1: Please provide the current title of manuscript

Manuscript ID no.: 6db7d35f-a66c-456e-98bd-beefe8e4bd3d

Title: The lncRNAs: Innovative Multifunctional Players of Drug Resistance in Colorectal Cancer

Section 2: Please provide the previous authorship, in the order shown on the manuscript before the changes were introduced. Please indicate the corresponding author by adding (CA) behind the name.

	First name(s)	Family name	ORCID or SCOPUS id, if available
1st author	Hossein	Maghsoudi	58693483500
2nd author	Farhad	Sheikhnia	https://orcid.org/0000-0002-3992-0088 57295253000
3rd author	Maryam	Abbastabar	57193336807
4th author	Bita	Azizzadeh	58039465300
5th author	Maryam	Majidinia	https://orcid.org/0000-0001-9776-5816 55392260500

Please use an additional sheet if there are more than 10 authors.

Section 3: Please provide a justification for change. Please use this section to explain your reasons for changing the authorship of your manuscript, e.g. what necessitated the change in authorship? Please refer to the (journal) policy pages for more information about authorship. Please explain why omitted authors were not originally included and/or why authors were removed on the submitted manuscript.

Subject: Author Position and Addition Updates for Manuscript

Dear Editors of Cancer Cell International,

I hope this message finds you well.

I am writing to inform you of some updates regarding the author positions and additions for our manuscript, as per the recent comments from reviewers which necessitated major revisions. These revisions included the addition of new sections and comprehensive proofreading.

SPRINGER NATURE ---

Springer Nature is one of the world's leading global research, educational and professional publishers, created in May 2015 through the combination of Nature Publishing Group, Palgrave Macmillan, Macmillan Education and Springer Science+Business Media.

SPRINGER NATURE Change of authorship request form - Journals (pre-acceptance)

We have enlisted the expertise of Motahareh Rasoulzadeh to contribute to the writing of new sections. Additionally, Ahmad Fazilat and Mohamad Valilo have assisted in editing and revising the tables, as well as proofreading the manuscript to ensure it adheres to appropriate English language standards, free from errors in syntax, grammar, and spelling.

Given their significant contributions, we have accordingly updated the positions of the authors and added the new contributors. The final order of authors is: Hossein Maghsoudi, Motahareh Rasoulzadeh, Maryam Abbastabar, Ahmad Fazilat, Farhad Sheikhnia, Bita Azizzadeh, Mohamad Valilo, and Maryam Majidinia.

Thank you for your understanding and consideration. We look forward to your positive feedback.

Best regards,

Maryam Majijina, Corresponding Author

Section 4: Proposed new authorship. Please provide your new authorship list in the order you would like it to appear on the manuscript. Please indicate the corresponding author by adding (CA) behind the name. If the Corresponding Author has changed, please indicate the reason under section 3.

	First name(s)	Family name (this name will appear in full on the final publication and will be searchable in various abstract and indexing databases)	Affiliated institute	E-mail address
1st author	Hossein	Maghsoudi	[1]Student Research Committee, Urmia University of Medical Sciences, Urmia, Iran. [2]Department of Clinical Biochemistry, School of Medicine, Urmia University of Medical Sciences, Urmia, Iran.	hoseinmaghsudy@yahoo.com
2nd author	Motahareh	Rasoulzadeh	[3]Department of Toxicology and Pharmacology, School of Pharmacy, Shahid Beheshti University of Medical Sciences, Tehran, Iran	Nzn.f1995@gmail.com
3rd author	Maryam	Abbastabar	[4]Department of Clinical Biochemistry, School of Medicine, Babol University of Medical Sciences, Babol, Iran.	Maryam.atbr1372@gmail.com

SPRINGER NATURE ---

Springer Nature is one of the world's leading global research, educational and professional publishers, created in May 2015 through the combination of Nature Publishing Group, Palgrave Macmillan, Macmillan Education and Springer Science+Business Media.

4th author	Ahmad	Fazilat	[5]Motamed Cancer Institute, Breast Cancer Research Center, ACECR, Tehran, Iran	jonhacopian@gmail.com
5th author	Farhad	Sheikhnia	[1]Student Research Committee, Urmia University of Medical Sciences, Urmia, Iran. [2]Department of Clinical Biochemistry, School of Medicine, Urmia University of Medical Sciences, Urmia, Iran.	Farhad.lab95@gmail.com
6th author	Bita	Azizzadeh	[6]Solid Tumor Research Center, Cellular and Molecular Medicine Institute, Urmia University of Medical Sciences, Urmia, Iran.	bitaazizzadeh1127@gmail.com
7th author	Mohamad	Valilo	[1]Student Research Committee, Urmia University of Medical Sciences, Urmia, Iran. [2]Department of Clinical Biochemistry, School of Medicine, Urmia University of Medical Sciences, Urmia, Iran.	valilo.biomed@gmail.com
8th author	Maryam	Majidinia	[6]Solid Tumor Research Center, Cellular and Molecular Medicine Institute, Urmia University of Medical Sciences, Urmia, Iran.	majidinia.m@umsu.ac.ir

Please use an additional sheet if there are more than 10 authors.

Section 5: Author contribution, Acknowledgement and Disclosures. Please use this section to provide a new disclosure statement and, if appropriate, acknowledge any contributors who have been removed as authors and ensure you state what contribution any new authors made (if applicable per the journal or book (series) policy). Please ensure these are updated in your manuscript - after approval of the change(s) - as our production department will not transfer the information in this form to your manuscript.

New acknowledgements:

None.

New Disclosures (financial and non-financial interests, funding):

Not Applicable.

SPRINGER NATURE ---

Springer Nature is one of the world’s leading global research, educational and professional publishers, created in May 2015 through the combination of Nature Publishing Group, Palgrave Macmillan, Macmillan Education and Springer Science+Business Media.

New Author Contributions statement (if applicable per the journal policy):
HM, MR, MA, AF, FS, and MV wrote the article and prepared the tables; BA prepared the figure; MM designed and revised the article. All the authors studied and approved the final manuscript. The authors declare that all data were generated in-house and that no paper mill was used.

State 'Not applicable' if there are no new authors.

Section 6: Declaration of agreement. *All* authors, unchanged, new and removed *must* sign this declaration.
(NB: Please print the form, (docu)-sign and return/upload a scanned copy. Please note that signatures that have been inserted as an image file are acceptable as long as it is handwritten. Typed names in the signature box are unacceptable.) *** Please delete as appropriate. Delete all of the bold if you were on the original authorship list and are remaining as an author.**

	First name	Family name		Signature	Date
1st author	Hossein	Maghsoudi	I agree to the proposed new authorship shown in section 4 /**and the addition/removal*of my name to the authorship list** /and the proposed change in corresponding author	Hossein Maghshoudi	Oct 11th, 2024
2nd author	Motahareh	Rasoulzadeh	I agree to the proposed new authorship shown in section 4 /**and the addition/removal*of my name to the authorship list** /and the proposed change in corresponding author	Motahareh Rasoulzadeh	Oct 11th, 2024

SPRINGER NATURE ---
Springer Nature is one of the world's leading global research, educational and professional publishers, created in May 2015 through the combination of Nature Publishing Group, Palgrave Macmillan, Macmillan Education and Springer Science+Business Media.

Change of authorship request form - Journals (pre-acceptance)

3rd author	Maryam	Abbastabar	I agree to the proposed new authorship shown in section 4 /**and the addition/removal*of my name to the authorship list** /and the proposed change in corresponding author	maryam abbastabar	Oct 11th, 2024
4th authors	Ahmad	Fazilat	I agree to the proposed new authorship shown in section 4 /**and the addition/removal*of my name to the authorship list** /and the proposed change in corresponding author	Ahmad Fazilat	Oct 11th, 2024
5th author	Farhad	Sheikhnia	I agree to the proposed new authorship shown in section 4 /**and the addition/removal*of my name to the authorship list** /and the proposed change in corresponding author	Farhad Sheikhnia	Oct 11th, 2024
6th author	Bita	Azizzadeh	I agree to the proposed new authorship shown in section 4 /**and the addition/removal*of my name to the authorship list** /and the proposed change in corresponding author	Bita Azizzadeh	Oct 11th, 2024
7th author	Mohamad	Valilo	I agree to the proposed new authorship shown in section 4 /**and the addition/removal*of my name to the authorship list** /and the proposed change in corresponding author	mohammad valilo	Oct 11th, 2024

SPRINGER NATURE ---

Springer Nature is one of the world's leading global research, educational and professional publishers, created in May 2015 through the combination of Nature Publishing Group, Palgrave Macmillan, Macmillan Education and Springer Science+Business Media.

SPRINGER NATURE

Change of authorship request form - Journals (pre-acceptance)

	First name	Family name		Signature	Date
8th author	Maryam	Majidinia	I agree to the proposed new authorship shown in section 4 **/and the addition/removal*of my name to the authorship list** /and the proposed change in corresponding author	Maryam majidinia	Oct 11th, 2024

Please use an additional sheet if there are more than 10 authors.

In case of author collaborations with formal agreement:

	Name of consortium/consortia	First name	Family name		Signature	Date
Representative/legal guarantor				I agree to the proposed new authorship shown in section 4 **/and the addition/removal*of my name to the authorship list** /and the proposed change in corresponding author		

Both added/removed authors should complete the information in the first table under Section 6.

---- End of form ----

SPRINGER NATURE ---

Springer Nature is one of the world's leading global research, educational and professional publishers, created in May 2015 through the combination of Nature Publishing Group, Palgrave Macmillan, Macmillan Education and Springer Science+Business Media.
